# Predictive value of spirometry in early detection of lung disease in adults: a cohort study

**DOI:** 10.3399/bjgpopen20X101059

**Published:** 2020-08-05

**Authors:** Lene Maria Ørts, Bodil Hammer Bech, Torsten Lauritzen, Janus Laust Thomsen, Niels Henrik Bruun, Anders Løkke, Annelli Sandbæk

**Affiliations:** 1 Department of Public Health, Section for General Medical Practice, Aarhus University, Aarhus, Denmark; 2 Department of Public Health, Research Unit for Epidemiology, Aarhus University, Aarhus, Denmark; 3 Department of Clinical Medicine, Research Unit for General Practice, Aalborg University, Aalborg, Denmark; 4 Department of Medicine, Lillebælt Hospital, Vejle, Denmark

**Keywords:** early diagnosis, primary health care, lung diseases, obstructive, spirometry

## Abstract

**Background:**

Spirometry is essential to identify cases with obstructive lung diseases (OLDs) in primary care. However, knowledge about the long-term prognostic outcome among younger individuals is sparse.

**Aim:**

To describe the predictive value of spirometry among individuals in the age groups 30–49 years and 45–64 years.

**Design & setting:**

A population-based cohort study supplied with data from Danish national registries.

**Method:**

Spirometry was performed in 905 adults aged 30–49 years in 1991 and in 1277 adults aged 45–64 years in 2006. The participants were categorised into three groups: forced expiratory volume in 1 second (FEV_1_)/forced vital capacity (FVC) <70, 70–75, and >75. They were followed throughout 2017 using Danish national registries. Lung disease was defined as fulfilling at least one of the following: two prescriptions for respiratory medicine were redeemed within a year; one lung-related contact to the hospital; or lung-related death.

**Results:**

In the 1991 cohort, 21% developed lung diseases and in the 2006 cohort 17% developed lung diseases throughout 2017. The probability of developing lung disease if FEV_1_/FVC 70–75 was 35% (95% confidence interval [CI] = 25% to 44%) in the 1991 cohort and 23% (95% CI = 17% to 28%) in the 2006 cohort. The positive predicted value (PPV) was higher for both cohorts when focusing on smoking history and self-reported respiratory symptoms.

**Conclusion:**

The initial spirometry has a high predictive value to identify cases of future lung diseases. In addition, the group with FEV_1_/FVC 70–75 had a high risk of developing lung diseases later in life, suggesting this group would be a meaningful target of special interest.

## How this fits in

Few studies have evaluated different approaches to case finding of OLDs. To the authors' knowledge, there are no published studies investigating the value of spirometry (especially FEV_1_/FVC 70–75) in adults to predict later development of lung disease. This study demonstrates that GPs should pay attention to people with FEV_1_/FVC 70–75, as well as those with FEV_1_/FVC <70.

## Introduction

The Danish Health Authority recommends that GPs offer spirometry testing to all individuals aged >35 years with at least one of the following symptoms: cough, dyspnoea, wheeze or sputum production, or relevant exposure (current smoker and/or occupational exposure) to facilitate early detection of lung disease.^1^

Spirometry is essential in identification of chronic obstructive pulmonary disease (COPD) and asthma. An initial spirometry measures FEV_1_ and FVC. A fixed FEV_1_/FVC <70 is considered as an abnormal lung function and is the preferred diagnostic method by the Global Initiative for Obstructive Lung Disease (GOLD) as well as GPs.^2^ However, this method can potentially lead to overdiagnosing among^3,4^ older people and underdiagnosing among younger individuals.^5,6^

In a previous study^7^ it was found that adults with FEV_1_/FVC 70–75 have a higher risk of receiving respiratory medicine, have more GP contacts, and a lower income compared with those with a normal spirometry, defined as FEV_1_/FVC >75. This poor prognosis emphasises the importance of exploring the value of identifying the group with a FEV_1_/FVC 70–75.

Thus, this study aimed to describe the predictive value of spirometry among individuals in the age groups 30–49 years (1991 cohort) and 45–64 years (2006 cohort).

## Method

### Population

This Danish cohort study was based on data from the population based Ebeltoft Health Promotion Project (EHPP) initiated in 1991.^8^ In September 1991, 905 adults aged 30–49 years from the municipality of Ebeltoft (1991 cohort) were included. The participants were followed from 1991 throughout 2017. During the 27 years of follow-up 88 participants from the 1991 cohort died. The registries used in this study were not established before 1995 and it was thus not possible to ensure that the population were in good respiratory health in 1991. A new round of health examinations were performed in Ebeltoft in 2006, where 1322 adults aged 45–64 years were included (2006 cohort). All the included participants underwent spirometry and filled in the questionnaire. From the 2006 cohort, 45 participants were excluded who redeemed prescribed respiratory medicine or had a lung-related hospital contact within a period of 3 years before the spirometry in 2006, leaving 1277 for follow-up. During the 11 years of follow-up 76 participants from the 2006 cohort died. In total, 573 participated in both cohorts.

**Figure 1.**
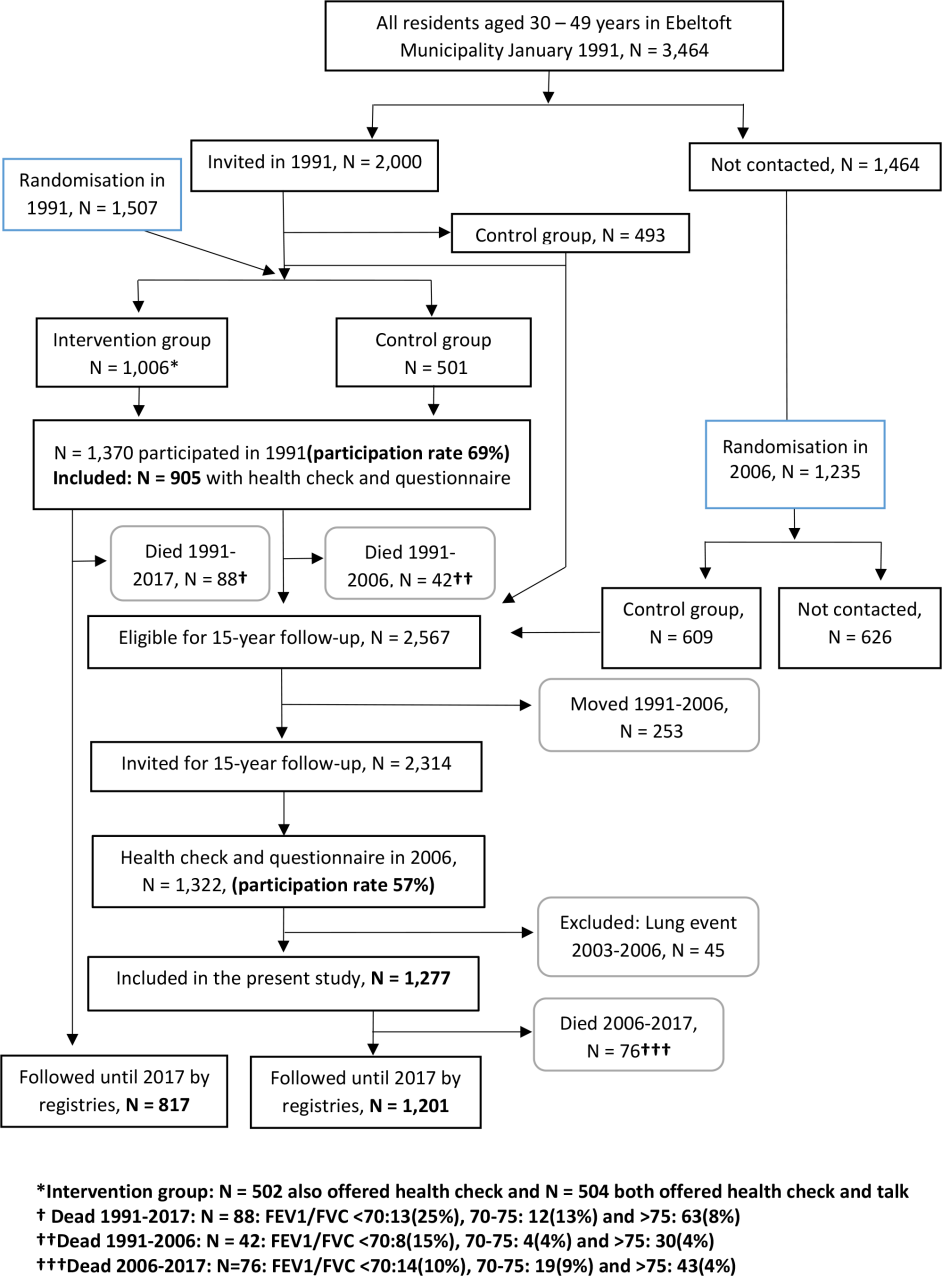
Flowchart of participant inclusion

Informed consent to participate was obtained by every participant before entering the EHPP.^8^ The design and the main flow of participants are shown in Figure 1, and are explained in detail elsewhere.^8^

### Exposure

The exposure of interest was the FEV_1_/FVC ratio. A direct-writing Vitalograph model R71 dry-wedge spirometer was used to measure lung function.^9^ The spirometer was calibrated in accordance with the guidelines.^10^ FEV_1_ and FVC were obtained from all participants based on at least three measurements differing by <5%. FEV_1_/FVC was categorised into three groups: <70, ≥70 to <75, or ≥75.

### Outcome

The outcome of interest was lung disease, which was defined as fulfilling at least one of the following criteria: redeeming two prescriptions for respiratory medicine within 1 year; one lung-related contact to the hospital; or lung-related death.

Danish registries were linked to clinical measurements using the 10-digit civil registration number (CPR number) assigned to all Danish residents.^11^ In Denmark, most medication, including respiratory medicine, is only available on prescription. Data concerning redeemed prescription medication were available from 1 January 1995 in the Danish Register of Medical Product Statistics.^12^ Respiratory medicine was defined as a prescription redeemed within the Anatomical Therapeutic Chemical (ATC) code R03 (drugs for OLDs).

Information on lung-related contacts to the hospital was extracted from the Danish National Patient Registry.^13^ In Denmark, the International Classification of Diseases version eight (ICD-8) was used until 1 January 1994 and followed by the ICD version 10 (ICD-10). Lung-related hospital contact was defined as having at least one hospital contact (outpatient or inpatient) with the following ICD codes: ICD-8: 162–163 lung cancer, 466 (bronchitis) and 490–493 (emphysema and asthma); and ICD-10: J20–23 (bronchitis), J40–47 (chronic lower respiratory diseases), and C34 and C78 (lung cancer).

Finally, data on lung-related death were obtained from The Danish Register of Causes of Death,^14^ which contains both the underlying cause of death and the condition or diagnosis that directly caused the death. Lung-related death was defined if either the direct cause of death or the underlying cause of death was coded with one of the following: J20–23 (bronchitis), J40–47 (chronic lower respiratory diseases), or C34 and C78 (lung cancer).

### Covariates

From the questionnaires completed in 1991 and 2006, respectively, information was obtained on age, sex, respiratory symptoms (subdivided into: no, light, or severe symptoms) and smoking (subdivided into: never, former, or current smoker). Data were collected on weight, height, body mass index (BMI), and lung function based on results from the clinical examinations in 1991 and 2006, respectively.

Information on sociodemographic profile was obtained from Statistics Denmark and measured at inclusion in 1991 and 2006.^15^ Cohabitation status was defined as cohabiting or living alone. Income level was defined using the family’s disposable Organisation for Economic Co-operation and Development (OECD)-adjusted income level (mean and tertiles). Finally, educational level was defined as the highest attained education and divided into three groups based on UNESCO categories.^16^

### Statistics

The characteristics of the baseline participants in 1991 and in 2006 were summarised using mean and standard deviations for continuous variables, and absolute frequency and percentage for categorical variables. The composite outcome was calculated for all participants and presented as event or no event. The predictive value of the spirometry test for participants with FEV_1_/FVC <70, ≥70 to <75, or ≥75 was calculated as the probability of a positive event during follow-up and presented with 95% CI. Subanalyses were performed for participants with a smoking history, reporting of respiratory symptoms, or both. For the subanalyses, smoking history was dichotomised into yes (current/former) or no (never), and respiratory symptoms was dichotomised into yes (severe/light) or no.

All analyses comply with the Danish regulations on registry-based research^17^ and all data were fully anonymised. All statistical analyses were performed using the Stata (version 14) software package (https://www.stata.com).

## Results

The overall number of participants included in the 1991 cohort and the 2006 cohort are shown in Table 1. In the 1991 cohort, the mean age was 40.3 years, 63.8% had a smoking history, and 20.4% reported respiratory symptoms within the last year before the health check. In the 2006 cohort, the mean age was 55.9 years, 47.5% had a smoking history, and 22.2% reported respiratory symptoms within the last year.

**Table 1.** Baseline characteristics of the 1991 cohort and the 2006 cohort

	**1991 cohort**	**Missing**	**2006 cohort**	**Missing**
Characteristics	**Total**	***n* / *N***	**Total**	***n* / *N***
***n* (** **%)**	905 (100.0)	0/905	1277 (100.0)	0/1277
**Sex, male, *n* (** **%)**	430 (47.5)	0/905	634 (49.6)	0/1277
**Age, mean (** **SD** **)**	40.3 (5.7)	0/905	55.9 (5.7)	0/1277
**Living alone, *n* (** **%)**				
Yes	175 (19.5)		260 (20.4)	
No	724 (80.5)	6/905	1017 (79.6)	0/1277
**Education, *n* (** **%)**				
Low (≤10 years)	249 (28.2)		322 (25.5)
Medium (>10 to ≤15 years)	435 (49.3)		613 (48.6)	
High (>15 years)	199 (22.5)	22/905	327 (25.9)	15/1277
**Income, 1000 euro, mean** **(** **SD** **)**	15.2 (5.0)	8/905	33.0 (25.2)	4/1277
**Income, 1000 euro, *n*** **(%)**				
Low tertile	316 (34.9)		423 (33.2)	
Middle tertile	310 (34.3)		425 (33.4)	
High tertile	279 (30.8)	0/905	425 (33.4)	4/1277
**Respiratory symptoms, previous year, *n* (** **%)**				
No symptoms	721 (79.7)		944 (77.8)	
Light symptoms	150 (16.6)		202 (16.7)	
Severe symptoms	34 (3.8)	0/905	67 (5.5)	64/1277
**Smoking status at baseline, *n*** **(%)**				
Never smoker	326 (36.2)		659 (52.5)	
Current smoker	461 (51.2)		392 (31.2)	
Former smoker	113 (12.6)	5/905	204 (16.3)	22/1277
**BMI, mean (** **SD** **)**	25.1 (4.3)	0/905	27.1 (4.6)	0/1277
**FEV**_**1**_ **/FVC, mean (** **SD** **)**	80.1 (6.2)	0/905	77.7 (7.3)	0/1277
**FEV**_**1**_ **% of predicted value, mean (SD)^a^**	98.2 (12.2)	0/905	93.7 (15.5)	0/1277
**FEV**_**1**_ **/FVC, *n* (%**)				
<70	52 (5.7)		138 (10.8)	
≥70 to <75	95 (10.5)		204 (16.0)	
≥75	758 (83.8)	0/905	935 (73.2)	0/1277

BMI = body mass index. FEV_1_/FVC = forced expiratory volume in 1 second/forced vital capacity. SD = standard deviation. ^a^The predicted value of FEV_1 _was calculated by taking the age, height, and reference values into account.

The present study showed that the predictive value was highest among individuals with FEV_1_/FVC <70 and lowest among FEV_1_/FVC >75. However, the probability of developing lung disease if FEV_1_/FVC 70–75 was 35% (95% CI = 25% to 44%) in the 1991 cohort, and 23% (95% CI = 17% to 28%) in the 2006 cohort when looking at the overall group (Table 2).

**Table 2.** Positive predictive value of a positive test calculated on the 1991 cohort and 2006 cohort

**Table 2** **A**	1991 cohort, **all**	2006 cohort, **all**
	Composite outcome 1991–2017, *n* (95% CI)	Composite outcome 2006–2017, *n* (95% CI)
	Event	No event	Total	Event	No event	Total
FEV_1_/FVC <70	27	25	52	56	82	138
FEV_1_/FVC 70–75	33	62	95	46	158	204
FEV_1_/FVC >75	131	627	758	115	820	935
Total	191	714	905	217	1060	1277
PPV <70		0.52 (0.38 to 0.66)		0.41 (0.32 to 0.49)
PPV 70–75		0.35 (0.25 to 0.44)		0.23 (0.17 to 0.28)
PPV >75		0.17 (0.15 to 0.20)		0.12 (0.10 to 0.14)
						
**Table 2B**	**1991 cohort, respiratory symptoms**			**2006 cohort, respiratory symptoms**		
	**Composite outcome** **1991** **–** **2017** **,** ***n*** **(95% CI)**			**Composite outcome 2006** **–** **20** **17** **,** ***n*** **(95% CI)**		
	Event	No event	Total	Event	No event	Total
FEV_1_/FVC <70	14	9	23	30	14	44
FEV_1_/FVC 70–75	19	12	31	18	32	50
FEV_1_/FVC >75	37	93	130	42	133	175
Total	70	114	184	90	179	269
PPV <70		0.61 (0.41 to 0.81)		0.68 (0.54 to 0.82)
PPV 70–75		0.61 (0.44 to 0.78)		0.36 (0.23 to 0.49)
PPV >75		0.28 (0.21 to 0.36)		0.24 (0.18 to 0.30)
**Table 2C**	**1991 cohort, smoking history**			**2006 cohort, smoking history**		
	**Composite outcome** **1991** **–** **2017** **, *n* (95% CI)**			**Composite outcome 2006** **–** **20** **17, *n* (95% CI)**		
	Event	No event	Total	Event	No event	Total
FEV_1_/FVC <70	23	21	44	37	49	86
FEV_1_/FVC 70–75	27	44	71	29	89	118
FEV_1_/FVC >75	88	371	459	61	331	392
Total	138	436	574	127	469	596
PPV <70		0.52 (0.38 to 0.67)		0.43 (0.34 to 0.52)
PPV 70–75		0.38 (0.27 to 0.49)		0.25 (0.17 to 0.32)
PPV >75		0.19 (0.16 to 0.23)		0.16 (0.12 to 0.19)
**Table 2D**	**1991 cohort, smoking history and respiratory symptoms**			**2006 cohort, smoking history and respiratory symptoms**		
	**Composite outcome** **1991** **–** **2017, *n* (95% CI)**			**Composite outcome 2006** **–** **20** **17, *n* (95% CI)**		
	Event	No event	Total	Event	No event	Total
FEV_1_/FVC <70	12	8	20	24	9	33
FEV_1_/FVC 70–75	15	11	26	12	21	33
FEV_1_/FVC >75	31	60	91	24	61	85
Total	58	79	137	60	91	151
PPV <70		0.60 (0.39 to 0.81)		0.73 (0.58 to 0.88)
PPV 70–75		0.58 (0.39 to 0.77)		0.36 (0.20 to 0.53)
PPV >75		0.34 (0.24 to 0.44)		0.28 (0.19 to 0.38)

FEV_1_/FVC = forced expiratory volume in 1 second/forced vital capacity. PPV = positive predictive value.

In subgroups of participants with self-reported respiratory symptoms and/or a smoking history (Table 2), the predictive value was even higher. Among the participants with a FEV_1_/FVC 70–75 and self-reported respiratory symptoms, the predictive value was 61% (95% CI = 44% to 78%) in the 1991 cohort and 36% (95% CI = 23% to 49%) in the 2006 cohort (Table 2). Focusing on the group with a smoking history and a FEV_1_/FVC 70–75, the predictive value was 38% (95% CI = 27% to 49%) in the 1991 cohort and 25% (95% CI = 17% to 32%) in the 2006 cohort (Table 2). The distribution of the composite outcome is shown in Table 3.

**Table 3.** Description of the composite outcome in the 1991 cohort (1991–2017) and the 2006 cohort (2006–2017)

	1991 cohort, *n* (%)		2006 cohort, *n* (%)
Positive composite outcome	191 (100)		217 (100)
Respiratory medicine	164 (85.9)		181 (83.4)
Lung-related contact to hospital	87 (45.5)		97 (44.7)
Lung-related death	16 (8.4)		18 (8.3)

## Discussion

### Summary

At a population level in both cohorts the FEV_1_/FVC ratio had a predictive value for development of lung disease later in life. As expected, the predictive value was highest among the group with a FEV_1_/FVC <70. However, the groups with a FEV_1_/FVC 70–75 also had applicable results. This supports the idea that a fixed cut-off of 70 among younger adults may underdiagnose OLD.^5^ By adding information about smoking history and self-reported respiratory symptoms as recommended,^1^ the PPV increased.

### Strengths and limitations

A major strength of this study is the high participation rate (69% in 1991 and 57% in 2006) in a real-life setting with a clinical examination, including spirometry performed both in 1991 and 2006.^8^ Additionally, the authors had the unique opportunity to make a complete and long-term registry-based follow-up from 1991 throughout 2017.

However, there are some limitations. First, there is a risk of selection bias. It is known from previous studies that on average non-attendees are less healthy and have a worse socioeconomic profile than attendees.^18,19^ Furthermore, 573 participants in 2006 had a previous health check, which potentially could have led to more health-related talks on health promotion and smoking cessation. This has probably resulted in an underestimation of the predictive value in the 2006 cohort, as participants had followed the advice and stopped smoking between the two clinical examinations in 1991 and 2006. Second, the composite outcome is based on information from Danish registries. Although highly validated, it is possible that not all individuals with a lung disease were identified. The authors do not have information on diagnostic spirometry examinations performed by GPs during follow-up; therefore, it is possible that some patients could have had a FEV_1_/FVC <70 and were recommended to quit smoking, but not prescribed any medical treatment. If this is the event, the predictive value has been underestimated. To increase the specificity of the composite outcome, the study only included at least two prescriptions redeemed for respiratory medicine within a year as an outcome, thereby excluding coincidental treatment prescribed for cough or flu. Finally, the spirometry measure used in this study is only pre-bronchodilator measurements, which may overestimate the prevalence of airflow limitation.^20^ Yet, in the present study, the risk of overestimating was substantially reduced by excluding the 45 individuals with known lung diseases 3 years prior to the clinical examination in 2006. Unfortunately, the opportunity to exclude individuals with lung diseases 3 years prior to the clinical examination in 1991 did not arise, which may have overestimated the PPVs in 1991.

The invited participants were from the general population in Ebeltoft Municipality, Denmark. Hence, the generalisability of the findings will not only mirror certain individuals at risk; for example, patients with lung symptoms or current smokers. The real-life setting provided high external validity, as the individuals are representative for subjects to be screened for OLD in primary care. Finally, life expectancy has increased during the follow-up period. Smoking rates have decreased and new inhalers have been developed and widely introduced, which may have affected the generalisability of the study.^21^ Smoking cessation and relevant medical treatment are the primary interventions to improve lung function and reduce the complication rate, which may help explain the difference between the baseline smoking prevalence in the two cohorts^22^ (Table 1).

### Comparison with existing literature

A variety of approaches to identify and diagnose individuals with OLD in primary care settings has been investigated.^23–30^ Guirguis-Blake *et al* ^27^ compared questionnaires and spirometry as a COPD screening instrument. They found a higher predictive value of the spirometry (PPV 63%–75%) compared with the COPD diagnostic questionnaire (PPV 17%–45%). The results from the present study support the findings by Guirguis-Blake *et al*, but the highest PPV was found when combining the spirometry result and self-reported respiratory symptoms.

Another screening instrument is the handheld spirometer, which has been tested in different settings such as pharmacies, market places, and general practice.^28–31^ Frith *et al* validated the handheld spirometry in a high-risk population and found a PPV of 73% with a fixed ratio cut-off of 70, and a PPV of 52% with a fixed ratio cut-off of 75.^29^ Thorn *et al* provided an initial handheld spirometry to all smokers when they visited the GP for other reasons and found that 25.2% had undiagnosed COPD.^28^ The study group also tested different cut-offs for fixed ratios when providing initial spirometry to a primary care population consisting of current smokers aged 45–85 years. The choice of use for the optimal fixed ratio cut-off was a FEV_1_/FVC < 73 where sensitivity was 79.2 and specificity was 80.3.^28^

Finally, the lower limit of normal (LLN) has been discussed as an alternative to the fixed ratio. However, clinicians favour the fixed ratio owing to simplicity and the GOLD recommendations,^2^ whereas the demand for accuracy is used among some pulmonary physiologists and researchers arguing for the LLN.^4,25,27^

The distribution of the FEV_1_/FVC values among adults and middle-aged people can be explained by the natural history of lung function.^32,33^ It is expected that the FEV_1_/FVC will reach a 'plateau phase' in the mid-20s followed by an incipient decline.^32,33^ On the contrary, Lange *et al* found that only one-fourth of 40 year olds with obstructive spirometry end up with COPD, which questions the physiological development of lung diseases.^34^

### Implications for research and practice

In the Danish healthcare system, primary care is free of charge and 98% of Danish citizens are assigned to a GP.^35^ The GPs conduct the case finding of OLD, perform the major part of the care, including medicine adjustments, and motivate smoking cessation. Therefore, primary care has a huge impact on the state of health of patients with OLD, since it is only the most severe cases that are referred to a pulmonary specialist. In the present study, most of the patients with a positive event were not hospitalised, indicating that their disease has been managed by their GP.

Further work is needed to improve the early case finding of lung diseases in primary care. First, the detection of those actually at risk should be enhanced. The individuals at risk are less prone to participate in health promotion,^18,19^ recommending that the GPs should have more focus on targeted case finding when patients visit the GP for other reasons. Although sensitivity and specificity of the handheld spirometry could be improved, there are several advantages to the simple and reliable tool. A handheld spirometry compared with the diagnostic spirometry is time-saving; 4 minutes 17 seconds versus 32 minutes 31 seconds, respectively. Thereby, the related costs amount to 2.12 EUR versus 16.07 EUR, respectively.^28^ The number needed to screen to detect one case of COPD is 4.6 and spirometry is cost-effective in the early identification of COPD.^26^ Still, there is a lack of spirometry use in general practice.^36^ The groups with a FEV_1_/FVC <70 had a PPV of 41% and 52%, which emphasises that less than half of patients with OLDs end up receiving medical treatment.

In conclusion, this descriptive study has shown a high risk of developing lung diseases later in life among those with FEV_1_/FVC 70–75. The findings support that early case finding of lung diseases is possible and suggests that both adults and middle-aged people with FEV_1_/FVC 70–75 should be a meaningful target of intervention as those with FEV_1_/FVC <70.

## References

[1] National Board of Health (2018). [Recommendations for patients with COPD] *Anbefalinger for tværsektorielle forløb for mennesker med KOL* (in Danish).

[2] Global Initiative for Chronic Obstructive Lung Disease (2020). 2020 Gold reports. https://goldcopd.org/gold-reports.

[3] Hardie JA, Buist AS, Vollmer WM (2002). Risk of over-diagnosis of COPD in asymptomatic elderly never-smokers. Eur Respir J.

[4] Bakke PS, Rönmark E, Eagan T (2011). Recommendations for epidemiological studies on COPD. Eur Respir J.

[5] Cerveri I, Corsico AG, Accordini S (2008). Underestimation of airflow obstruction among young adults using FEV1/FVC <70% as a fixed cut-off: a longitudinal evaluation of clinical and functional outcomes. Thorax.

[6] Levy ML, Quanjer PH, Booker R (2009). Diagnostic spirometry in primary care: proposed standards for general practice compliant with American Thoracic Society and European Respiratory Society recommendations. Prim Care Respir J.

[7] Ørts LM, Bech BH, Lauritzen T (2020). Lung function in adults and future burden of obstructive lung diseases in a long-term follow-up. NPJ Prim Care Respir Med.

[8] Lauritzen T, Leboeuf-Yde C, Lunde IM, Nielsen KD (1995). Ebeltoft project: baseline data from a five-year randomized, controlled, prospective health promotion study in a Danish population. Br J Gen Pract.

[9] Vitalograph (2019). Welcome to Vitalgraph International. https://vitalograph.eu.

[10] Moore VC (2012). Spirometry: step by step. Breathe.

[11] Schmidt M, Pedersen L, Sørensen HT (2014). The Danish Civil Registration System as a tool in epidemiology. Eur J Epidemiol.

[12] Kildemoes HW, Sørensen HT, Hallas J (2011). The Danish National Prescription Registry. Scand J Public Health.

[13] Lynge E, Sandegaard JL, Rebolj M (2011). The Danish National Patient Register. Scand J Public Health.

[14] Helweg-Larsen K (2011). The Danish register of causes of death. Scand J Public Health.

[15] Thygesen LC, Daasnes C, Thaulow I, Brønnum-Hansen H (2011). Introduction to Danish (nationwide) registers on health and social issues: structure, access, legislation, and archiving. Scand J Public Health.

[16] Jensen VM, Rasmussen AW (2011). Danish education registers. Scand J Public Health.

[17] Statistics Demark (2015). Guidelines for transferring aggregated results from Statistics Denmark’s research services. https://www.dst.dk/en/TilSalg/Forskningsservice.

[18] Ørts LM, Løkke A, Bjerregaard A-L (2019). The effect on participation rates of including focused spirometry information in a health check invitation: a cluster-randomised trial in Denmark. BMC Public Health.

[19] Bjerregaard A-L, Maindal HT, Bruun NH, Sandbæk A (2017). Patterns of attendance to health checks in a municipality setting: the Danish 'Check Your Health Preventive Program'. Prev Med Rep.

[20] Johannessen A, Omenaas ER, Bakke PS, Gulsvik A (2005). Implications of reversibility testing on prevalence and risk factors for chronic obstructive pulmonary disease: a community study. Thorax.

[21] Pisinger C, Jørgensen T, Toft U (2018). A multifactorial approach to explaining the stagnation in national smoking rates. Dan Med J.

[22] Scanlon PD, Connett JE, Waller LA (2000). Smoking cessation and lung function in mild-to-moderate chronic obstructive pulmonary disease. the lung health study. Am J Respir Crit Care Med.

[23] Haroon S, Adab P, Griffin C, Jordan R (2013). Case finding for chronic obstructive pulmonary disease in primary care: a pilot randomised controlled trial. Br J Gen Pract.

[24] Fisk M, McMillan V, Brown J (2019). Inaccurate diagnosis of COPD: the Welsh national COPD audit. Br J Gen Pract.

[25] Oh DK, Baek S, Lee SW (2018). Comparison of the fixed ratio and the *Z*-score of FEV__1__/FVC in the elderly population: a long-term mortality analysis from the Third National Health and Nutritional Examination Survey. Int J Chron Obstruct Pulmon Dis.

[26] Løkke A, Ulrik CS, Dahl R (2012). Detection of previously undiagnosed cases of COPD in a high-risk population identified in general practice. COPD.

[27] Guirguis-Blake JM, Senger CA, Webber EM (2016). Screening for chronic obstructive pulmonary disease. JAMA.

[28] Thorn J, Tilling B, Lisspers K (2012). Improved prediction of COPD in at-risk patients using lung function pre-screening in primary care: a real-life study and cost-effectiveness analysis. Prim Care Respir J.

[29] Frith P, Crockett A, Beilby J (2011). Simplified COPD screening: validation of the PiKo-6® in primary care. Prim Care Respir J.

[30] Castillo D, Guayta R, Giner J (2009). COPD case finding by spirometry in high-risk customers of urban community pharmacies: a pilot study. Respir Med.

[31] Haroon SM, Jordan RE, O'Beirne-Elliman J, Adab P (2015). Effectiveness of case finding strategies for COPD in primary care: a systematic review and meta-analysis. NPJ Prim Care Respir Med.

[32] Fletcher C, Peto R (1977). The natural history of chronic airflow obstruction. Br Med J.

[33] Løkke A, Marott JL, Mortensen J (2013). New Danish reference values for spirometry. Clin Respir J.

[34] Lange P, Celli B, Agustí A (2015). Lung-function trajectories leading to chronic obstructive pulmonary disease. N Engl J Med.

[35] Andersen JS, Olivarius NDF, Krasnik A (2011). The Danish National Health Service Register. Scand J Public Health.

[36] Koefoed MM, dePont Christensen R, Søndergaard J, Jarbøl DE (2012). Lack of spirometry use in Danish patients initiating medication targeting obstructive lung disease. Respir Med.

